# Detection of Circulating Parasite-Derived MicroRNAs in Filarial Infections

**DOI:** 10.1371/journal.pntd.0002971

**Published:** 2014-07-17

**Authors:** Lucienne Tritten, Erica Burkman, Andrew Moorhead, Mohammed Satti, James Geary, Charles Mackenzie, Timothy Geary

**Affiliations:** 1 Institute of Parasitology, Centre for Host-Parasite Interactions, McGill University, Sainte-Anne-de-Bellevue, Quebec, Canada; 2 Department of Infectious Diseases, College of Veterinary Medicine, University of Georgia, Athens, Georgia, United States of America; 3 Filariasis Research Reagent Resource Center, College of Veterinary Medicine, University of Georgia, Athens, Georgia, United States of America; 4 Department of Pathobiology and Diagnostic Investigation, College of Veterinary Medicine, Michigan State University, East Lansing, Michigan, United States of America; National Institutes of Health, United States of America

## Abstract

Filarial nematodes cause chronic and profoundly debilitating diseases in both humans and animals. Applications of novel technology are providing unprecedented opportunities to improve diagnosis and our understanding of the molecular basis for host-parasite interactions. As a first step, we investigated the presence of circulating miRNAs released by filarial nematodes into the host bloodstream. miRNA deep-sequencing combined with bioinformatics revealed over 200 mature miRNA sequences of potential nematode origin in *Dirofilaria immitis*-infected dog plasma in two independent analyses, and 21 in *Onchocerca volvulus*-infected human serum. Total RNA obtained from *D. immitis*-infected dog plasma was subjected to stem-loop RT-qPCR assays targeting two detected miRNA candidates, miR-71 and miR-34. Additionally, *Brugia pahangi*-infected dog samples were included in the analysis, as these miRNAs were previously detected in extracts prepared from this species. The presence of miR-71 and miR-34 discriminated infected samples (both species) from uninfected samples, in which no specific miRNA amplification occurred. However, absolute miRNA copy numbers were not significantly correlated with microfilaraemia for either parasite. This may be due to the imprecision of mf counts to estimate infection intensity or to miRNA contributions from the unknown number of adult worms present. Nonetheless, parasite-derived circulating miRNAs are found in plasma or serum even for those species that do not live in the bloodstream.

## Author Summary

Filarial parasites commonly infect humans and animals, especially in tropical settings. The strongly debilitating panel of diseases they cause in humans contributes to an entrenched cycle of poverty. For efficient treatment strategies, reliable diagnostic tests are necessary. We investigated the potential of parasite-derived microRNAs (miRNAs; short non-coding RNA molecules present in eukaryotes) as biomarkers of infection. Using deep-sequencing technologies and bioinformatics, we identified over two-hundred mature miRNA candidates of nematode origin in plasma from *Dirofilaria immitis*-infected dogs. Similarly, we discovered twenty-one miRNA candidates predicted to be released by *Onchocerca volvulus* in infected human sera. We developed two RT-qPCR assays for the detection of *D. immitis* miR-71 and miR-34 in dog plasma that discriminated infected from uninfected samples. We demonstrated the presence of filarial miRNAs in host blood, regardless of localization in their respective hosts, and suggest that they are suitable targets for detection by RT-qPCR.

## Introduction

Parasitic filarial nematodes cause significant public health problems in tropical and subtropical regions of the globe, to which their transmission by insect vectors is currently restricted. Over 150 million people are affected by filarial infections, the most common being lymphatic filariasis (caused by *Wuchereria bancrofti*, *Brugia malayi*, and *Brugia timori*) and onchocerciasis (caused by *Onchocerca volvulus*) [1]. These infections cause severe physical disability, chronic suffering and tremendous economic loss, thus contributing to an entrenched cycle of poverty [1], [2]. On the veterinary side, the dog heartworm *Dirofilaria immitis* is the most prevalent and problematic filarial parasite. Heartworm infection has been described on all continents, with its incidence increasing in tropical climates. As is the case for some other filariids, transmission occurs via a mosquito vector, occasionally and accidentally infecting humans [3].

Different filariid species and developmental stages occupy various tissues in their hosts, and therefore, diagnosing these infections remains challenging. Because *O. volvulus* microfilariae (mf) can be detected in the epidermis, skin snips have widely been employed for diagnostic purposes, despite reported sensitivity issues and the fact that skin-snipping is an unpopular, painful and invasive procedure [1], [4]. Drug-based patch tests are currently preferred, but are not quantitative. Antigen- or antibody-based diagnostic methods have been proposed but are not yet widely adopted [5]. However, even antigen or serological tests pose problems of suboptimal sensitivity or specificity [6]–[8]. Diagnosis of canine dirofilariosis relies on microscopic identification of circulating mf in the bloodstream, or adult antigen detection through a variety of commercially-available tests. However, mf may be absent from dog blood for several reasons (drug treatment, age of adult worms, etc.), and imperfect sensitivity is a concern with antigen detection methods [3]. Polymerase chain reaction (PCR)-based methods are the most accurate diagnostic approach for dirofilariosis to date [9], [10], but are not routinely employed. A major issue with currently available filarial diagnostic tests is their inability to predict adult worm burden and viability. This is an area in which improved techniques could help identify hosts in need of treatment and could be used to accurately quantitate treatment efficacy for both established and candidate antifilarial drugs.

MicroRNAs (miRNAs), first discovered in the free-living nematode *Caenorhabditis elegans*, are an abundant class of endogenous small (18–25 nucleotides), non-protein-coding RNA generally present in eukaryotes. miRNAs play important regulatory roles in gene expression by binding to messenger RNA (mRNA), primarily resulting in mRNA degradation and suppression of translation. The degree of complementarity between the mRNA 3′ untranslated region and the miRNA sequence determines the fate of the target transcript [11]–[13]. Most miRNAs are of intergenic origin, and are first transcribed within a long primary miRNA that is subsequently processed in the nucleus into a precursor miRNA. Precursor miRNAs adopt a hairpin conformation and are transported to the cytoplasm to undergo further enzymatic processing. Mature miRNAs exist as single-stranded molecules in the RNA-induced silencing complex (RISC) [12].

Circulating miRNAs have been detected in human fluids such as urine, blood and plasma and in mammalian cell culture medium [14]–[16]. Circulating miRNAs are found either in exosomes or in a protein-bound form (i.e., within RISC) [17], [18] and are generally thought to be involved in intercellular communication [19], [20].

The discovery of disease-specific miRNA profiles in the blood of patients with cancer, metabolic disorders or viral infections has raised great interest in their potential as diagnostic and prognostic biomarkers [21]–[24]. In many conditions such as cancer and inflammatory diseases, the presence in perturbed amounts of defined miRNA populations in different biological fluids reflects the pathophysiological status and the tissue affected [20], [25], [26].

In recent years, hundreds of nematode miRNAs have been described, many with key functions in development [12], [27], [28]. However, growing evidence implies a potential role for miRNAs in host-pathogen interactions and immune regulation [25], [29], or, as suggested by studies in *C. elegans*, as modulators of drug sensitivity [30], [31]. Circulating miRNAs from the trematode parasites *Schistosoma japonicum* and *S. mansoni* have recently been investigated [32], [33]. In *S. mansoni*-infected humans, three parasite-derived miRNAs (bantam, miR-277 and miR-3479-3p) were amplified and distinguished egg-positive from egg-negative individuals [33].

To our knowledge, this work represents the first description of circulating miRNAs from nematode parasites in host plasma and serum. Microfilariae of both *D. immitis* and *B. pahangi* are found in host blood, while *D. immitis* adults reside in the pulmonary artery of the heart, and *B. pahangi* adults inhabit lymphatic vessels. In contrast, *O. volvulus* life-cycle stages do not have direct contact with host blood. After identifying over 200 candidate nematode miRNAs in *D. immitis*-infected dog plasma and 21 candidate *O. volvulus* miRNAs in serum from infected patients, we determined whether parasite-derived mature miRNA sequences are detectable by an amplification approach.

## Materials and Methods

### Ethics statement

The onchocerciasis blood samples were collected as part of other studies conducted under a National Institutes of Health (USA) grant for International Collaborations in Infectious Disease Research and approved by the University Committee for Research Involving Human Subjects of Michigan State University. Samples were anonymized with a code that was used independent of the patient's identification. All animal procedures were approved by the University of Georgia, Institutional Animal Care and Use Committee, and complied with U.S. Department of Agriculture's regulations (animal utilization permits A2010 11-567 and A2010 12-005).

### Human samples

Serum samples were obtained from Sudanese onchocerciasis patients by venipuncture as part of a study conducted in 1985 [34]. Microfilarial counts in skin snips taken from eleven patients varied from 1.6 to 43.0 mf/mg, and included two sowda patients displaying loads of 0 and 0.6 mf/mg, respectively. Samples were transported to Michigan State University in liquid nitrogen and were stored at −80°C from the time of arrival. Eleven samples, ranging from 0.2 to 1.2 ml, were pooled for this analysis.

### Animals

Eleven dogs, infected with either *D. immitis* (n = 5) or *B. pahangi* (n = 2), or uninfected (n = 4), were used in this study. They were maintained at the Filariasis Research Reagents Resource Center (Athens, GA, USA). Dogs were inoculated subcutaneously with either 400 *B. pahangi* (FR3 strain) or 30 or 50 *D. immitis* L3 (MP3 and Missouri strains, respectively), or naturally-infected with *D. immitis* and hookworms (one dog). Mf counts were determined for each animal by a standard thick blood smear of 20 µl of blood, run in duplicate. A drop of water was added to lyse red blood cells and the smear was spread to a surface area of 1.5×2.5 cm for uniformity between slides. The number of mf between the two slides was averaged to obtain the mf count. Reported mf counts are an average of counts obtained over several months. Microfilaraemic peripheral blood was drawn from the jugular vein using standard procedures, and then placed in RNase-free EDTA tubes (for 10 ml blood: 100 µl EDTA 0.5 M, pH 8 (Thermo Scientific)).

### Total RNA purification, miRNA deep-sequencing and data analysis

Between 7.5 and 10 ml plasma was obtained from each control and infected dog (*D. immitis* or *B. pahangi*) by centrifugation of 20 ml peripheral blood at 1250× *g* for 15 min at 4°C. Total RNA, including small RNAs, was isolated from each sample using a Plasma/Serum Circulating RNA Purification Maxi Kit (Norgen Biotek, Canada), following the manufacturer's instructions. Eluted RNA was quantified by optical density (NanoDrop 1000, Thermo Scientific) and stored in aliquots at −20°C.

A pool of total RNA (1.1 µg) from the four *D. immitis* laboratory-infected dogs (134 ng from Dim1, 112 ng from Dim2, 196 ng from Dim3 and 554 ng from Dim4) was shipped on dry ice to LC Sciences (Houston, TX, USA) for miRNA deep sequencing and bioinformatics analysis. The rest of the isolated RNA was stored at −20°C for analysis by RT-qPCR. Subsequently, 3.2 µg total RNA were isolated in the same way from 30 ml plasma obtained from a single naturally-infected dog (≈35,000 mf/ml) co-infected with hookworms (10 eggs per gram), and sent for sequencing and bioinformatics analysis to LC Sciences as described above.

Total RNA was purified from the pool of onchocerciasis serum samples using the same kit and procedure described above, and 0.88 µg total RNA were sent to LC Sciences for miRNA sequencing and bioinformatics analysis.

The workflow included the production of a small RNA library using the Illumina TruSeq Small RNA Preparation kit (Illumina Inc., San Diego, CA, USA), following the Illumina TruSeq Small RNA Sample Preparation Guide (Illumina Inc., San Diego, CA, USA). Clusters were generated using the purified cDNA library on Illumina's Cluster Station and then sequenced on Illumina GAIIx following the manufacturer's instructions. Real-time sequencing image analysis and base-calling were performed using Real-Time Analysis version 1.8.70 (Illumina Inc., San Diego, CA, USA) and the raw 40 nt reads were retrieved with Sequencing Control Studio software version 2.8 (Illumina Inc., San Diego, CA, USA) and used for subsequent data analysis.

An LC Sciences proprietary pipeline script (ACGT101-miR v4.2) was employed for data analysis, with which raw data were filtered into mappable reads, subsequently mapped to the dog genome (*Canis lupus familiaris*) (ftp://ftp.ncbi.nlm.nih.gov/genomes/Canis_lupus_familiaris, accessed 03/14/2012) and to mature and precursor miRNA sequences of *Canis lupus familiaris* and other selected mammalian species, available from miRBase v19.0 and v20.0 [35]–[38] (ftp://mirbase.org/pub/mirbase/CURRENT/). The remaining unmapped reads were filtered against mRNA, the Rfam database (collection of RNA families) and RepBase (repetitive DNA elements database) [39], [40] (ftp://ftp.ncbi.nlm.nih.gov/genomes/Canis_lupus_familiaris/RNA, http://www.girinst.org/repbase, http://rfam.janelia.org). Finally, all reads unmapped to the dog genome were aligned to the *D. immitis* genome v2.2 [41] and selected nematode miRNAs (*Caenorhabditis elegans*, *Haemonchus contortus*, *Ascaris suum*, *Caenorhabditis remanei*, *Caenorhabditis briggsae*, *Pristionchus pacificus* and *Brugia malayi*), available from miRBase. The same procedure was followed for human and *O. volvulus*-derived miRNA prediction. The databases employed for this aspect were ftp://ftp.ncbi.nih.gov/genomes/H_sapiens/ and ftp://ftp.sanger.ac.uk/pub/pathogens/Onchocerca/volvulus/.

### Primer design for RT-qPCR experiments

Five highly abundant mature nematode-derived miRNA sequences (bma-miR-100d_R+1, bma-miR-100c_R+1_1ss12CT, asu-miR-71, cel-miR-34-5p_R+1_1ss1AT, and bma-miR-228), were selected for amplification by RT-qPCR. The sequences were mapped to *D. immitis*. At least 4 mismatches distinguished the chosen miRNA candidates from the closest dog or vertebrate miRNA homologue identified using miRBase and BLASTn [42]. One host miRNA (cfa-miR-223, not present in *D. immitis* or *B. pahangi*) served as control [43], [44]. An optimized stem-loop RT-qPCR protocol for miRNAs was employed for primer design and experiments [45]. HPLC-purified synthetic mature miRNA sequences and HPLC-purified oligonucleotides (stem-loop RT primers, forward primers, universal reverse primer) and hydrolysis Taqman probes were from Life Technologies (Life Technologies, Foster City, USA). The sequences are given in Tables S7, S8, S9. All oligonucleotides were reconstituted in Ultrapure water (GIBCO) and stored at −20°C pending use.

### Reverse transcription quantitative polymerase chain reaction

Ten-fold dilutions of synthetic miRNAs ranging from 10^8^ to 10^4^ molecules per RT reaction were prepared freshly in Ultrapure water (GIBCO) before each experiment to create standard curves [45].

Reverse-transcription was performed with a Maxima Universal First Strand cDNA synthesis kit (Thermo Scientific, # FERK1661) following the manufacturer's instructions. Reagents were freshly mixed before each experiment in a total volume of 10 µl, containing 0.5 µl 10 mM dNTPs, 100 units Maxima reverse transcriptase (except in RT – control tubes), 2 µl 5× buffer, 10 units RNase inhibitor, 3.75 µl nuclease-free H_2_O, 1.5 µl RT stem-loop primer (5 nM stock solution), and 1.25 µl miRNA template (sample or synthetic standard). Every step was performed on ice. The reaction was carried out in an Eppendorf Thermal Cycler (Mastercycler ep gradient, Eppendorf, Hamburg, Germany) at 65°C for 30 min followed by 85°C for 5 min before being held at 4°C. The resulting cDNA was used on the same day in qPCR reactions and the remaining RT product was stored at −80°C. In each RT-qPCR experiment, one RT reaction was performed per sample (and synthetic miRNA concentration). Experiments were carried out, three times independently, testing all dog samples in the same run.

Amplifications were performed in a total volume of 20 µl containing 1.3 µl fresh RT product and the Luminaris Probe qPCR colorless master mix (Thermo Scientific, # FERK0952), containing a hot start polymerase and uracil DNA glycosylase. A 20× mixed oligonucleotide stock was freshly prepared and the final concentrations described by Kramer [45] were strictly followed (specific forward primer: 1.5 µM; universal reverse primer: 0.7 µM; specific hydrolysis Taqman probe: 0.8 µM). cDNA from each sample was run in duplicate with a single RT- control. We used a RotorGene RG6000 apparatus (Corbett Research, Australia) with cycling conditions of 2 min at 50°C, 10 min at 95°C, followed by 55 cycles of denaturation at 95°C for 15 sec and annealing at 60°C for 1 min. Each experiment was performed three times independently, each time using different cDNA preparations from the same samples.

### Data analysis

Standard curves were generated by the software Rotor-Gene 6 (Corbett Research) based on the concentrations of synthetic miRNA. The automatic threshold function was applied to determine the threshold cycle (Ct) for each sample, and calculate the absolute number of copies with standard deviations. Data were normalized to the initial plasma volume. Because miR-223 levels might have been affected by different degrees of hemolysis in each plasma sample, the data were normalized to the total RNA quantity (measured by optical density) after purification, for miR-223 only.

## Results

### Sequencing of nematode miRNAs from canine plasma

We obtained 15,378,851 raw reads from the pooled *D. immitis*-infected dog plasma. After removal of low-quality sequences (junk, wrong size, etc.) and sequences mapping to the dog and other reference mammalian genomes (listed in Table S1), the remaining 4,430,628 reads were examined in more detail (Table 1). Sequences in this set corresponding to other RNA populations (e.g. mRNA, repetitive sequences, etc.) and those not corresponding to any miRBase entry or reference nematode genome were subtracted. The remaining sequences were clustered into eight groups after mapping to reference nematode genomes and filtering with secondary structure criteria. In total, 30764 unique sequences were identified. Ignoring group 4b, which consists of sequences that are unlikely to represent true nematode miRNAs, 245 miRNA candidates of potential nematode origin were detected in the infected dog plasma pool, with 75 miRNAs present in 10 copies or more (Table S2).

**Table 1:** Unique sequence clusters.

Clustering of 4,430,628 reads used for identification of *D. immitis* circulating miRNAs					
	Cluster definition	Raw reads	% total reads	Unique sequences	Comment
No hit	Sequences not predicted to be miRNAs or other RNA populations	2,789,147	62.95	NA	fraction discarded
Other RNA	Sequences corresponding to other RNA populations (mRNA, repetitive elements, other non-coding RNAs)	35,370	0.80	NA	fraction discarded
**Group 1a**	Sequences map to *C. elegans* miRNAs and *C. elegans* precursor mapped to the *D. immitis* genome with a similarity of >80%	0	0.00	0	
**Group 1b**	Sequences map to other nematode miRNAs and map to the *D. immitis* genome	338,694	7.63	45	
**Group 2a**	Sequences map to other nematode miRNAs and only mature (not precursor) sequences map to the *D. immitis* genome	31,441	0.71	31	
**Group 2b**	Same criteria as for group 2a, but the *D. immitis* genome sequences may not form hairpins	15,080	0.34	8	
**Group 3a**	Sequences map to other nematode miRNAs, but not to the *D. immitis* genome	439	0.01	24	
**Group 3b**	Same criteria as for group 3a, but sequences present other differences (i.e., >1 mismatch or mapped to other species)	30	<0.01	15	
**Group 4a**	Sequences do not map to other nematode precursor miRNAs but do map to *D. immitis* genome. Extended sequences may form hairpins.	32,721	0.74	122	may contain *D. immitis*-specific miRNAs
**Group 4b**	Sequences do not map to other nematode miRNAs and may not form hairpins in *D. immitis* mapping genome regions	1,187,706	26.81	30520	unlikely to be *D. immitis* miRNAs; cluster ignored in subsequent analyses.

4,430,628 reads were distributed into 8 clusters based on miRBase search, genome mapping and secondary structure criteria.

Only 45 of the 30764 unique sequences could be mapped to Nematoda miRNAs in miRBase (minus *C. elegans*) and to the *D. immitis* genome (Group 1b). Thirty-one unique sequences mapped to Nematoda miRNAs in miRbase. The mapped pre-miRNAs did not map to the *D. immitis* genome, but the mature sequences did (Group 2a). As many as 122 unique sequences did not map to Nematoda miRNAs in miRBase but mapped to regions of the *D. immitis* genome (Group 4a). This large group may contain potentially novel *D. immitis* miRNA sequences. Finally, 99.2% of the unique sequences did not map to Nematoda miRNAs in miRBase, but could be mapped to the *D. immitis* genome in regions which cannot form hairpins and thus are not likely to represent authentic miRNAs (Group 4b). Among Group 4a, only one predicted candidate (PC-5p-268380_2, Table S2) was reported in the previously published heartworm miRNA collection [46]. Table 2 shows the 10 most abundant miRNAs of probable nematode origin discovered in this experiment. Isoforms of miR-100 were the most abundant, followed by miR-71, miR-34, miR-228, miR-50, and miR-57. These mature sequences were reported previously in whole-worm extracts [46]. Two predicted candidates, not previously reported (PC-3p-132_7253 and PC-3p-208_4425), were present in >4000 copies and ranked among the top ten most abundant nematode miRNA candidates in infected dog plasma.

**Table 2:** Ten most abundant circulating nematode miRNAs in dog plasma.

	miRNA name [46]	representative miRNA	mature sequence	copy #
**1**	**miR-100d**	bma-miR-100d_R+1	TACCCGTAGCTCCGAATATGTGT	122967
**2**	**miR-100a**	bma-miR-100a_R+1	AACCCGTAGTTTCGAACATGTGT	99366
**3**	**miR-100c**	bma-miR-100c_R+1_1ss12CT	AACCCGTAGAATTGAAATCGTGT	21069
**4**	**miR-71**	bma-miR-71_R+4	TGAAAGACATGGGTAGTGAGACG	14707
**5**	**miR-34**	cel-miR-34-5p_R+1_1ss1AT	TGGCAGTGTGGTTAGCTGGTTGT	12928
**6**	**miR-228**	bma-miR-228	AATGGCACTAGATGAATTCACGG	10818
**7**	**miR-50**	bma-miR-50	TGATATGTCTGATATTCTTGGGTT	8814
**8**	**ND**	PC-3p-132_7253	TCCCGGCTCGTGGCACCAAATAGA	7253
**9**	**miR-57**	bma-miR-57	TACCCTGTGGTACCGAGCTGTGTCT	7026
**10**	**ND**	PC-3p-208_4425	GAATTCCTCTGCGGTAGTTACTGGA	4425

ND: not described.

Separately, miRNAs from a *D. immitis* naturally-infected dog concomitantly harboring a hookworm infection (determined *ex post facto*) were sequenced (Table S3). In groups 1b to 4a, 261 non-canine miRNA candidates were identified in this dog, with 79 sequences present in ≥10 copies. Among those 79, 87.3% were also found in the *D. immitis*-laboratory infected sample pool. The remaining 12.7% of these sequences were unique to the co-infected dog sample and might be derived from hookworms. Table S4 contains the unknown predicted candidates (Group 4a) found in both sequencing datasets.

### Sequencing of nematode miRNAs in serum from *O. volvulus*-infected humans

After removal of human and other mammalian sequences (Table S5), the pooled human onchocerciasis sample provided 2,179,119 high-quality reads that were clustered in groups, as described for *D. immitis*, and analyzed further. The largest fraction of reads (99.6%) was not predicted to be miRNAs or any other RNA, and 0.1% of the reads were classified as other RNA populations (mRNA, repetitive elements or other small RNAs). Only 21 unique sequences segregated in groups 1b to 4a and hence are legitimate candidate miRNAs (Table S6). A single unique sequence mapped to reference nematode miRNA homologues in miRBase and to the *O. volvulus* genome (Group 1b); another sequence mapped to *O. volvulus*, but the extended genome sequence from this locus is not predicted to form hairpins (Group 2b); Eleven unique sequences mapped to nematode miRNAs in miRBase, but did not map to the *O. volvulus* genome (Group 3a and 3b); Eight unique sequences found pre-miRNA nematode homologues in miRBase (but not mature miRNAs), and mapped to the *O. volvulus* genome (Group 4a). Another 310 sequences belonging to Group 4b were not taken into account for further analysis, as they combined lack of nematode homologs with the inability of the genomic region to form hairpins.

### miRNA amplification from plasma samples

Six miRNA candidate sequences were chosen for assay by RT-qPCR using total RNA from dog plasma. Assays were developed for two nematode miRNAs (see Table 2; miR-71 and miR-34 names assigned by homology to other nematode species), while repeated assays for three others (miR-100d, miR-100c, and miR-228) failed at the step of assay design despite extensive optimization attempts. An additional assay was developed for a dog control miRNA, cfa-miR-223.

Both miR-71 and miR-34 were detected in all four samples from dogs infected with *D. immitis* (Table 3). As miR-71 and miR-34 were previously reported in *B. pahangi* whole-worm extracts [28], we attempted their amplification from two *B. pahangi*-infected dogs. Both miR-71 and miR-34 were detected in *B. pahangi*-infected dog plasma. In contrast, neither miR-71 nor miR-34 was detected in samples from four uninfected dogs. miR-223 was detected in all samples, independent of infection status. Total RNA measured by optical density for each sample varied up to 5.1-fold, probably due to different degrees of hemolysis. This variability led us to normalize the qPCR data of endogenous miR-223 to sample ODs.

**Table 3:** Number of positive RT-qPCR reactions for miR-71, miR-34 and miR-223.

Positive detection on total reactions performed by RT-qPCR					
			nematode miRNAs		dog miRNA
	Sample	mf/ml	miR-71	miR-34	miR-223
**Uninfected**	**A**	-	0/6	0/6	6/6
	**B**	-	0/6	0/6	6/6
	**C**	-	0/6	0/6	6/6
	**D**	-	0/6	0/6	6/6
***Dirofilaria immitis***	**Dim1**	39200	6/6	6/6	6/6
	**Dim2**	40575	6/6	6/6	6/6
	**Dim3**	37700	6/6	6/6	6/6
	**Dim4**	96000	6/6	6/6	6/6
***Brugia pahangi***	**Bpa1**	20075	6/6	6/6	6/6
	**Bpa2**	43100	6/6	6/6	6/6

For each dog, the microfilaria (mf) counts per ml blood are given.

**Figure 1:**
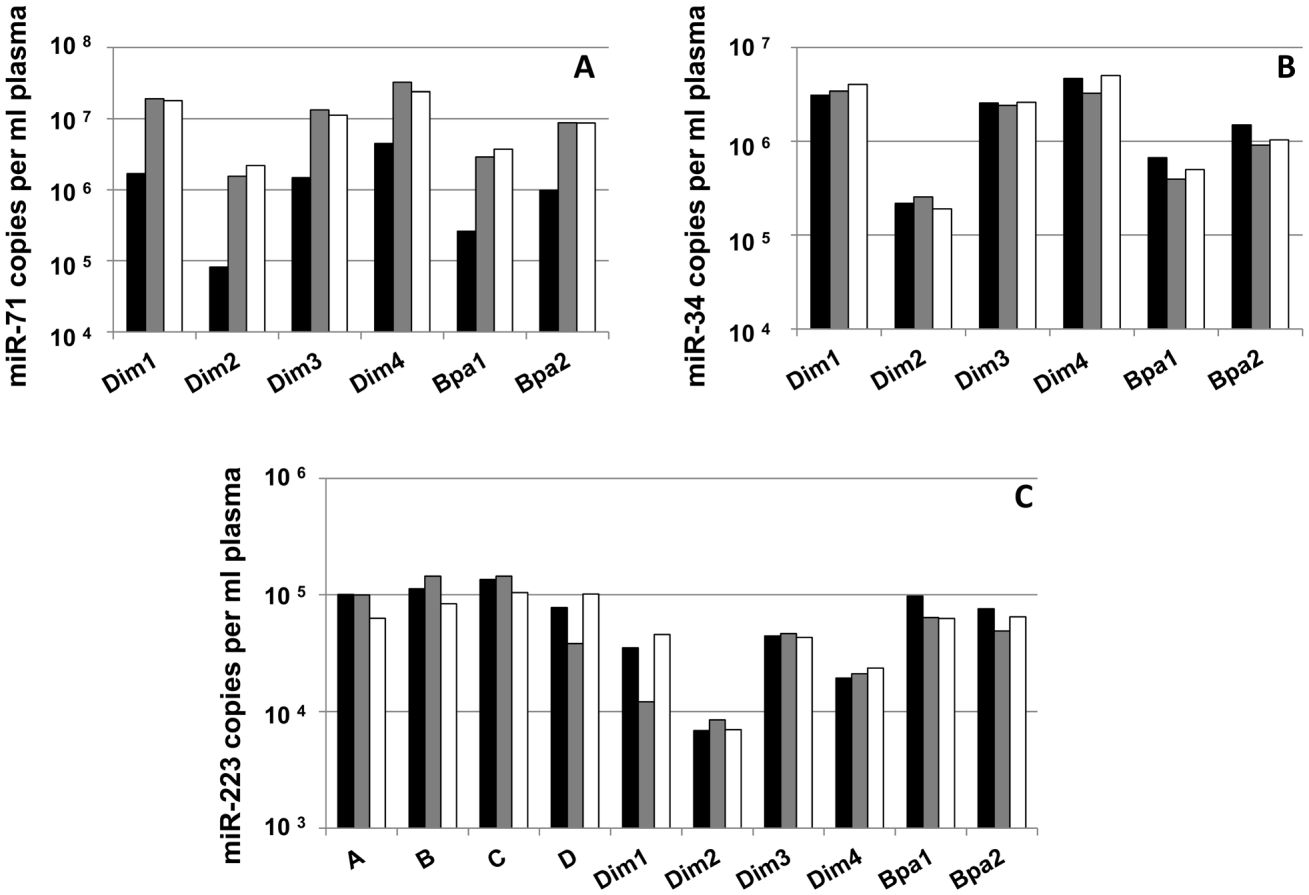
Extrapolated absolute miRNA copy number per ml plasma, from 3 independent RT-qPCR experiments. A: miR-71, only *D. immitis* and *B. pahangi*-infected samples; B: miR-34, only *D. immitis* and *B. pahangi*-infected samples; C: miR-223, all samples. Uninfected dogs = samples A–D. Black bars = experiment 1; gray bars = experiment 2; white bars = experiment 3.

**Figure 2:**
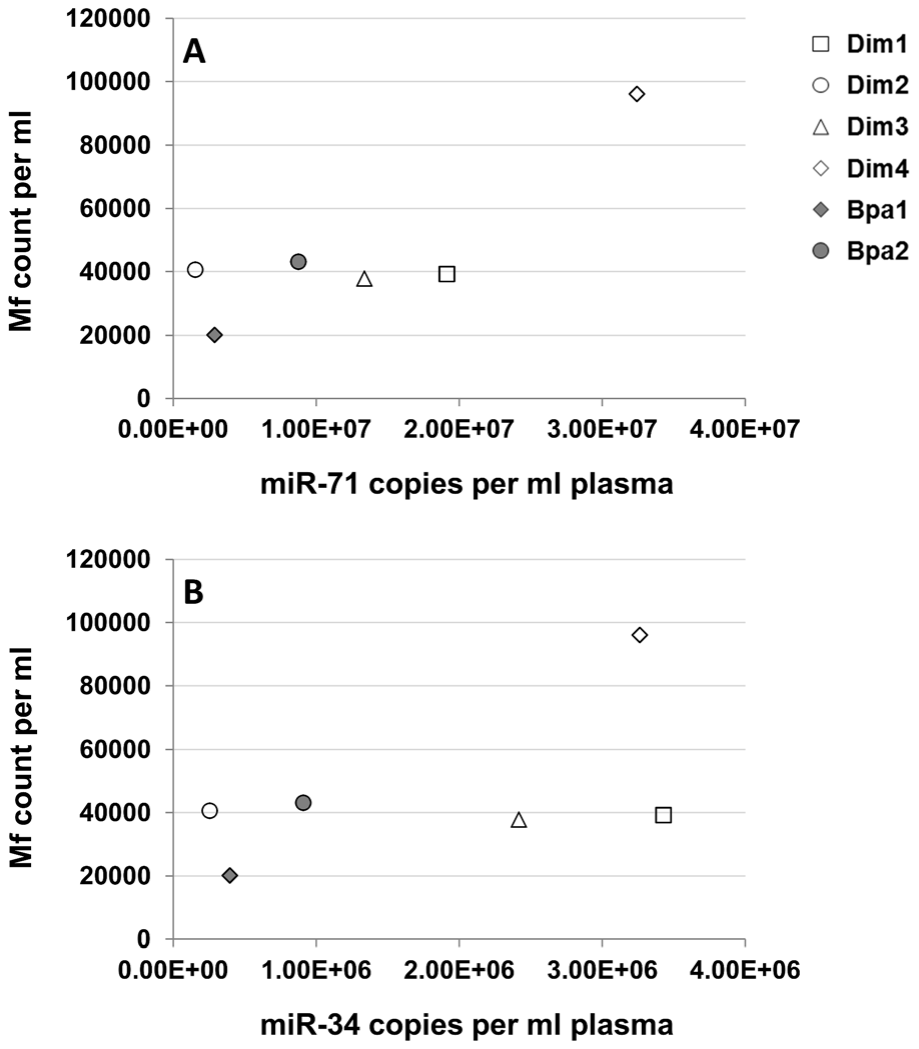
Relationship between miRNA copies per ml plasma and microfilaria counts. A: miR-71; B: miR-34. Empty symbols represent *D. immitis-* and filled symbols stand for *B. pahangi*-infected samples.

Copy number quantitation in each sample relied on the standard curve method (Figure 1). The most abundant of the detected miRNAs was miR-71, followed by miR-34 and miR-223. Cycle threshold (Ct) values and qPCR efficiencies are given in Tables S7, S8, S9. The greatest degree of inter-experimental variation was observed for miR-71, for which the absolute copy number per ml plasma varied by up to 27-fold between independent experiments using the same sample (Dim2). In contrast, the maximum copy number variation between experiments using the same sample was 1.7-fold for miR-34 and 3.8-fold for miR-223. Sample Dim4 always contained the highest amounts of miR-71, followed by Dim1, Dim3, Bpa2, Bpa1 and Dim2 in this order. The sample ranking was highly similar for miR-34, with Dim4 and Dim1 alternating with the highest copy numbers. If some associations could be recognized among samples containing miR-223 (i.e., sample C contained the highest amounts and Dim2 the least), the samples Dim1 and D showed high inter-experimental variation. Figure 2 shows the relationship between mf counts per ml dog blood (*D. immitis* and *B. pahangi*) and miR-71 and miR-34 copy numbers (from one experiment only; gray bars in Figure 1) per ml dog plasma. No correlation was observed between mf load and the abundance of either miRNA by RT-qPCR. Samples Dim1, Dim2, Dim3 and Bpa2 presented similarly high mf counts (∼40,000 mf/ml) but had miR-71 levels ranging from 1.5*10^6^ to 1.9*10^7^, a >12-fold difference. Similarly, >13-fold difference in miR-34 levels (2.5*10^5^–3.4*10^6^ copy number per ml plasma) was observed for the same four samples showing comparable mf counts.

## Discussion

Circulating miRNAs have been an increasing focus of diagnostic research for monitoring human diseases [47]. In the context of helminth infections, parasite-derived miRNA profiling in the host is in its infancy. So far, two publications have reported the presence of a few *Schistosoma* spp. miRNAs in plasma from infected rabbits, mice and humans [32], [33]. We identified a large number of candidate circulating miRNAs in samples of mammalian hosts infected with several species of filarial nematodes. We found 245 and 261 candidate miRNAs in independent samples of plasma obtained from heartworm-infected dogs. Ninety-six of these short sequences were present in both samples, almost entirely among the sub-set present in ≥10 copies. This strong concordance suggests that our methods were reliable and that these sequences represent authentic heartworm miRNAs. Divergence between the independent samples for low-abundance sequences is likely due to sensitivity of the methods; we cannot rule out based on these data the possibility that low abundance sequences also represent authentic circulating heartworm miRNAs.

Among the >1,000 miRNAs reported in adult heartworm extracts [46], only 21 were detected in dog plasma, 8 of them among the most abundant miRNAs detected in two independent experiments. The excellent agreement between two independent dog plasma samples (a pool of four laboratory-infected samples and a naturally-infected dog) suggests that at least 35 candidate miRNAs found in both datasets represent unreported *D. immitis* miRNAs (Table S4). The most abundant miRNA families we found to show considerable overlap with whole-worm sequences, with the most abundant miRNAs in adult worm extracts also detected in dog plasma; the miR-100d, miR-71, and miR-228 families ranked among the most abundant miRNAs [46]. The miR-100 group was represented by 11 predicted members in our study, three of which were found in >20,000 copies. In *C. elegans*, the *mir-51/mir-100* family is involved in several events, including embryogenesis, growth, male mating and pharyngeal attachment [48]. miR-228 is one of the most highly abundant families observed in *D. immitis* adults, with no known function. We detected three predicted miR-228 family members, while Fu and colleagues [46] reported only one. Neither miR-50 nor miR-57 was among the most abundant sequences in adult worm extracts. A proposed function for *mir-57* in *C. elegans* is in posterior cell fate determination, patterning the tail shape [49]. No function for miR-50 in nematodes has been proposed.

miR-71 is present in several helminth species [28], [50], [51]. Among nematodes, the mature sequence identified in *D. immitis* is completely conserved in *A. suum*, *B. pahangi* and *C. elegans* [28], [46], [52], [53], while the sequence is truncated in *B. malayi* [54] and *H. contortus* [28]. Proposed functions of miR-71 include positive influence on lifespan, as demonstrated in *C. elegans*; mutants bearing a deletion of *miR-71* were short-lived and less resistant to oxidative and heat stress [55], [56]. In *C. elegans*, both miR-71 and miR-34 showed stage-specific up-regulation in dauer larvae compared to late second-stage larvae [57].

The miR-34 family has been described in a number of species. The mature sequence is 100% identical in *D. immitis*, *B. malayi*, *B. pahangi*, and *A. suum*, but differs slightly in *C. elegans* and *Trichinella spiralis* [28], [46], [52]–[54], [58]. Like miR-71, miR-34 promotes longevity in *C. elegans* [55]. In terms of absolute copy numbers, miR-34 was approximately 12 times more abundant in infected dog plasma than in the whole adult extract [46]. Inversely, miR-1, the most abundant miRNA found in adults, was detected in only 5 copies in our study. A possible explanation is that miR-34 may be released in higher proportions by mf than by adults. The relationship between miR-71 or miR-34 expression levels and mf counts tended to follow a similar pattern.

We lack information on the adult worm burden in the dogs used in this study, and stage and sex-dependent influences on the release of specific miRNAs have not been reported. The poor correlation between mf counts and miRNA copy numbers suggests that adults could play a significant role in the release of the different miRNA candidates. However, mf counts are known to undergo daily and seasonal periodicity [59], and therefore are not considered reliable measurements of infection intensity. In addition, the duration and turnover of circulating miRNAs in plasma are unknown.

In humans, miR-223 plays a pivotal role in inflammation and infection, especially in granulocyte differentiation processes, and their subsequent activation pattern [60]. Although miR-223 was reported to increase in rabbit serum upon *S. japonicum* infection [61], we found no evidence for a similar change in heartworm-infected dogs. However, these elements make cfa-miR-223 an inappropriate endogenous control for normalization of RT-qPCR data in a context of infection. A challenge in experimental design where multiple species are involved is to identify species-specific (and fairly distant from potential homologues) miRNA sequences for amplification, and hence discernment between species. In addition, multiplex assays, as opposed to independent reactions for each miRNA, are desirable in order to generate true technical controls.

The *O. volvulus*-infected pooled sera sample revealed 21 *O. volvulus*-derived miRNA candidates and <1,000 known or predicted human miRNA sequences. This is slightly fewer than the ∼1,200 known or predicted canine miRNA sequences discovered in the fresh *D. immitis*-infected dog pooled plasma samples. However, the relative abundance of human miRNAs in the human serum pool was about 10-fold less than the abundance of canine miRNAs in the heartworm samples (not shown), suggesting that RNA degradation over time in storage likely reduced our ability to detect *O. volvulus* miRNAs in this experiment. Nevertheless, the much lower number of *O. volvulus* miRNA candidates detected and their low abundance could also be explained in part by the fact that this parasite does not reside in the bloodstream. Finally, plasma vs. serum samples were analyzed, further complicating comparison between the species [47], [62].

None of the 21 *O. volvulus* candidate miRNAs had obvious homologues in the *D. immitis* datasets. That *O. volvulus* apparently releases different miRNA populations might reflect its different habitat and consequent differences in parasite physiology. Additional analyses of freshly obtained samples would resolve some of these issues. Their possible utility as diagnostic targets would be enhanced if abundance is higher in fresh samples.

The mechanism of release of nematode miRNAs into the host bloodstream is unknown. Although it is not possible to rule out worm death or disintegration of some mf during processing of blood samples, a possibility is that nematodes secrete microvesicles containing miRNAs, together with proteins [63]. Microvesicles (i.e., exosomes) are considered the main miRNA carriers between communicating cells [20], and have been described in parasitic trematodes [64].

In the present work, we developed two RT-qPCR assays capable of detecting *D. immitis* and *B. pahangi* infections, after RNA isolation from plasma samples. Since mature sequences are often identical or very close among nematodes, the proposed assays may not be able to differentiate infection with other species (e.g., *A. suum*). Further efforts to validate *D. immitis*/*B. pahangi* miR-71 and miR-34 as diagnostic candidates are necessary, as the resulting copy numbers displayed extreme variation. A clear characterization of the nature of infection, including adult worm burden and sex ratio, is needed to complement mf counts, and to better understand the observed variation in abundance of circulating miRNAs. Technical limitations could occur in cases of low-intensity infections, and therefore, the minimal detection level must be defined for each miRNA target, and correlated with parasite counts. Using RT-qPCR, femtomolar concentrations of miRNAs can be detected [65], [66]. The observed high variation in total RNA recovery from a standard volume of plasma is likely to be due to different degrees of hemolysis in our samples. Most host serum miRNAs are directly derived from blood cells [67]. This, in turn, is likely to have biased the endogenous miR-223 levels [47], [68]. Therefore, RNA purification methods should be chosen and standardized carefully. Although the reagents are costly compared to assays with similar sensitivity, the stem-loop RT-qPCR presents a combination of high sensitivity and specificity, ease of operation and a requirement for relatively basic laboratory equipment. Moreover, multiplex amplification assays could be developed by using different dyes.

To the best of our knowledge, this is the first report of circulating miRNAs from *D. immitis* and *O. volvulus* in plasma and serum from their respective hosts. We found >200 mature miRNA candidate sequences of *D. immitis*-origin in infected dog plasma in two independent sequencing performances, and 21 mature miRNA candidate sequences in infected human serum, predicted to be released by *O. volvulus*. RT-qPCR assays have been developed and shown to efficiently discriminate *D. immitis*-infected (or *B. pahangi*-infected) versus uninfected samples, however without reflecting mf counts and with yet poorly reliable miRNA copy quantification. This study shows that filarial miRNAs are present in plasma and serum, even when the parasites do not reside in blood.

## Supporting Information

Table S1 Dog miRNA candidate sequences identified by deep-sequencing.
(XLSX)

Table S2 *Dirofilaria immitis* miRNA candidate sequences identified by deep-sequencing.
(XLSX)

Table S3 *D. immitis* miRNA candidate sequences identified by deep-sequencing from a co-infection with hookworm.
(XLSX)

Table S4 Overlapping unknown *D. immitis* predicted candidates (group 4a) between both datasets.
(XLSX)

Table S5 Human miRNA candidate sequences identified by deep-sequencing.
(XLSX)

Table S6 *Onchocerca volvulus* miRNA candidate sequences identified by deep-sequencing.
(XLSX)

Table S7 Detailed RT-qPCR information for miR-71.
(XLSX)

Table S8 Detailed RT-qPCR information for miR-34.
(XLSX)

Table S9 Detailed RT-qPCR information for miR-223.
(XLSX)
